# Vegan-mycoprotein concentrate from pea-processing industry byproduct using edible filamentous fungi

**DOI:** 10.1186/s40694-018-0050-9

**Published:** 2018-04-02

**Authors:** Pedro F. Souza Filho, Ramkumar B. Nair, Dan Andersson, Patrik R. Lennartsson, Mohammad J. Taherzadeh

**Affiliations:** 1 Swedish Centre for Resource Recovery, University of Borås, 50190 Borås, Sweden; 2 Mycorena AB, Stena Center 1A, 411 92 Gothenburg, Sweden; 3 Faculty of Caring Science, Work Life and Social Welfare, University of Borås, 50190 Borås, Sweden

**Keywords:** Pea-processing byproduct, Edible filamentous fungi, Vegan-mycoprotein concentrate, Meat substitute

## Abstract

**Background:**

Currently around one billion people in the world do not have access to a diet which provides enough protein and energy. However, the production of one of the main sources of protein, animal meat, causes severe impacts on the environment. The present study investigates the production of a vegan-mycoprotein concentrate from pea-industry byproduct (PpB), using edible filamentous fungi, with potential application in human nutrition. Edible fungal strains of Ascomycota (*Aspergillus oryzae*, *Fusarium venenatum*, *Monascus purpureus*, *Neurospora intermedia*) and Zygomycota (*Rhizopus oryzae*) phyla were screened and selected for their protein production yield.

**Results:**

*A. oryzae* had the best performance among the tested fungi, with a protein yield of 0.26 g per g of pea-processing byproduct from the bench scale airlift bioreactor cultivation. It is estimated that by integrating the novel fungal process at an existing pea-processing industry, about 680 kg of fungal biomass attributing to about 38% of extra protein could be produced for each 1 metric ton of pea-processing byproduct. This study is the first of its kind to demonstrate the potential of the pea-processing byproduct to be used by filamentous fungi to produce vegan-mycoprotein for human food applications.

**Conclusion:**

The pea-processing byproduct (PpB) was proved to be an efficient medium for the growth of filamentous fungi to produce a vegan-protein concentrate. Moreover, an industrial scenario for the production of vegan-mycoprotein concentrate for human nutrition is proposed as an integrated process to the existing PPI production facilities.

## Background

Attributed to the rise in population, urbanization, and income, a steady growth in the consumption of protein from animal sources has been observed in developed countries over the last few decades [1]. Nevertheless, currently around one billion people in the world do not have access to a diet which provides enough protein and energy [2]. Lack of protein can result in severe health problems such as growth failure, muscle weakness and an impaired immune system. Protein-energy malnutrition (PEM) can lead to conditions such as kwashiorkor and marasmus. Additionally, the production of meat has a heavy impact on the environment and makes a large contribution to the eutrophication process [3, 4]. In this context, it is important to find an alternative, cheap, and less resource-consuming protein source to substitute meat or meat products. Fungal organisms such as mushrooms and truffles have traditionally been part of human nutrition largely because of their flavor; however they cannot be considered as an important source of protein in comparison to meat-based sources [1, 5]. Considerable attention has been recently given to the use of filamentous fungi as a commercial human food component; especially due to their high protein content with all the essential amino acids to human nutrition, easy digestibility, low-fat content (cholesterol free), and the presence of dietary fibers [6]. The fibre content (6% w/w) is also comparable with other vegetarian protein sources [7].

Several strains of edible filamentous fungi have been recognized as a traditional source of palatable food by many societies around the globe, especially in Asia [8]. *Rhizopus* sp. has been used for centuries in the oriental cuisine in the preparation of fermented food such as tempeh [9]. *Aspergillus oryzae* also has culinary applications for the production of hamanatto, miso and shoyu. *Neurospora intermedia* is used in the preparation of the Indonesian staple food oncom [6]. Similarly, *Monascus purpureus* has been used as coloring and flavoring agent in food and beverages, as in the production of red yeast rice and rice wine [10–12]. Other applications of filamentous fungi include the production of several ingredients for the food and beverage industries, especially enzymes. In recent years, production of vitamins and polyunsaturated fatty acids by these microorganisms has been receiving increased attention [13]. Single-cell protein (SCP) can also be produced by filamentous fungi. An example currently in the market is the filamentous fungus *Fusarium venenatum*, commercialized under the name Quorn™. The fungus is cultivated in a synthetic medium with glucose, ammonium and supplemented with biotin. The costs associated with the substrate and the lack of competition results in a market price for the SCP that is higher than that of meat. Despite the high price, the fungal SCP has found its place in the market as a healthy substitute to meat, with its presence only in developed markets such as Europe and USA [6, 14]. Nevertheless, the SCP from the mycelium of filamentous fungi can be inexpensively produced when using cheap materials as substrates [15]. One such example is the pea processing industry byproduct that is being used in the present study.

Pea (*Pisum sativum*) is the second most important leguminous crop in the world with an annual production above 17 million metric tons, finding its applications mainly as food and feed. Originally from western Asia and northern Africa, its production has spread to over 10 million hectares of farmlands, especially in Russia, China, Canada, Europe, Australia and the United States. Rich in protein, carbohydrate, dietary fiber, vitamins, and minerals, the peas are used to produce food ingredients such as proteins, starches, flours, and fibers [16–18]. Pea proteins have faced a growth in food applications due to their nutritional and functional benefits, including their balanced amino acid profile, positive fat- and water-binding capabilities, emulsification and gelation properties, texture, and nutritional values. Moreover, allergies to pea are less frequent than allergies to other protein-rich grains, like soy [19]. Pea proteins have also been demonstrated as a useful ingredient in the formulation of antihypertensive foods because of their antihypertensive effects [16, 19, 20].

Pea proteins are commercialized in three forms: pea flour, pea protein concentrate, and pea-protein isolate (PPI). The manufacturing of pea flour consists of the dry milling of hulled peas, whereas pea protein concentrate is obtained by dry separation techniques. PPI production generally occurs by isoelectric precipitation at pH around 4.5, followed by a membrane separation technique to increase the protein concentration, such as ultrafiltration and diafiltration. PPI can be used in the preparation of dairy-based beverages, sports and nutritional foods, and other non-dairy sports products, such as vegan style yogurts. Additionally, it can partially replace dairy protein in therapeutic beverages and powders [19, 20]. Despite the high quality of the protein, the pea-processing byproduct (PpB) is considered to have poor functional properties. Therefore, its uses in food applications are limited and it is mainly produced as a byproduct of the protein extraction process [21]. A novel and alternative approach to valorize this pea-processing byproduct (PpB), as discussed in the present study is to convert it into a vegan-mycoprotein concentrate for human food applications, using edible strains of filamentous fungi. Intake of mycoprotein may be beneficial to human health [7, 22–24]. Several studies have investigated the cholesterol-lowering effects of mycoprotein; the results from these studies point to the same direction with reductions in both total and LDL-cholesterol [7, 22]. The greatest benefits are seen in individuals with a higher cholesterol level at baseline and in hypercholesterolaemic subjects. There is a difference between the macronutrients regarding satiety; protein is generally recognised as most satiating, followed by carbohydrates and fat [25]. Compared to other protein sources such as chicken, mycoprotein seems to be more satiating and hence have the possibility to decrease energy intake in subsequent meals [7, 23, 24]. It is possible that fibres in mycoprotein, one-third chitin and two-thirds beta-glucan, might have a specific effect on satiety [7]. Additionally mycoprotein appears to affect the glycaemic response positively [7, 24]. The exact mechanism that explains this is not known, but might be associated with its fibre content [7].

The aim of the present study was hence to convert PpB, a cheap and low-nutritional value byproduct of the PPI production, into a vegan-mycoprotein concentrate for human food applications. Five strains of filamentous fungi, namely *A. oryzae*, *F. venenatum*, *M. purpureus*, *N. intermedia* and *R. oryzae*, were screened for their growth to maximize the protein yield from the PpB. The best cases of the fungal growth were selected and further scaled-up in a bench airlift bioreactor, considering the industrial application potential of the process.

## Methods

### Substrate and enzymes

Pea-processing byproduct (PpB) used for this study was kindly provided by Protein Consulting AB (Sweden). The powder was sieved through a pore size of 0.2–0.25 mm and was characterized using triplicate samples for its carbohydrate, protein, ash, and moisture content. Cellulase cocktail Cellic Ctec2 (Novozymes, Denmark) with 94 FPU/mL activity at 35 °C, amyloglucosidase from *Aspergillus niger* (300 U/mL activity at 35 °C), and α-amylase from *Aspergillus oryzae* (100 U/mg activity at 35 °C) were supplied by Sigma-Aldrich Co. (Germany).

### Microorganisms

Edible food-grade filamentous fungi were used in the present study. The Ascomycota strains were *Neurospora intermedia* CBS 131.92 (Centraalbureau voor Schimmelcultures, Netherlands), *Aspergillus oryzae* var. *oryzae* CBS 819.72, *Monascus purpureus* CBS 109.07, and *Fusarium venenatum* ATCC 20334 (American Type Culture Collection, USA), and the Zygomycota strain was *Rhizopus oryzae* CCUG 61147 (Culture Collection University of Gothenburg, Sweden). All the fungal cultures were maintained on potato dextrose agar (PDA) slants containing (in g/L) potato extract 4; glucose 20; agar 15 and were renewed every 6 months. New PDA plates were prepared via incubation for 3–5 days at 30 °C followed by storage at 4 °C. For preparing spore solution, PDA plates (72 h grown) were flooded with 20 mL sterile distilled water and the spores were released by gently agitating the mycelium with a disposable cell spreader. An inoculum of 3 mL spore suspension (with a spore concentration of 3.9 × 10^5^–3.8 × 10^6^ spores/mL) per liter of the medium was used for the cultivations, unless otherwise specified. For preparing fungal biomass inoculum, the spores were inoculated into 100 mL YPD broth containing (in g/L) glucose 20, peptone 20, and yeast extract 10. The culture was incubated aerobically for 48 h at 35 °C and 125 rpm. The fungal biomass was harvested at the end of the cultivation and used as the inoculum. Dry weight was determined by drying at 105 °C overnight.

### Experimental set-up for fungal cultivation

The cultivations in 250 mL Erlenmeyer flasks were carried out using 100 mL of culture medium consisting only of the PpB substrate dissolved in distilled water. The optimum concentration of the medium was determined by testing the maximum load of PpB substrate which could go through the sterilization operation without causing retrogradation. The term retrogradation refers to the changes in the gelatinized starch during cooling, when the molecules recrystallize. This process leads to the production of a starch with high resistance to the enzymatic attack, thus reducing the effectiveness of the microbial metabolism [26–28]. The Erlenmeyer flasks were kept in a water bath shaker at 35 °C and 150 rpm (with a 9 mm orbital shaking radius). The pH of the sample was adjusted to 5.5 ± 0.1 prior to autoclaving by adding HCl 1 M. The enzymes were added according to the substrate at a load of 150 U/g for α-amylase, 163 U/g for amyloglucosidase, and 24 FPU/g for cellulase. At the end of the cultivations, the produced fungal mycelium was collected using a sieve, washed with ultra-pure water, dried at 70 °C and had the weight and protein content analysed. Samples were taken during cultivation to follow the consumption of sugars and the production of metabolites. All the cultivation experiments were conducted in duplicate and the mean values are presented with standard deviations.

### Scaling-up of the fungal cultivation in a bench scale airlift reactor

A 4.5-L airlift bioreactor (Belach Bioteknik, Sweden) with a working volume of 3.5 L was used to scale-up the fungal cultivation process. The entire bioreactor and the draft tube were made of transparent borosilicate glass. An internal loop with cylindrical geometry with 58 mm of diameter, 400 mm of height and 3.2 mm in thickness was used to achieve the airlift-liquid circulation. Aeration at the rate of 0.42 v.v.m. (volume_air_/volume_media_/min) was maintained throughout the cultivation, using a sintered stainless steel air-sparger with a pore size of 90 μm. Filtration of the inlet air was achieved by passing it through a polytetrafluoroethylene (PTFE) membrane filter (0.1 μm pore size, Whatman, Florham Park, NJ, USA). The cultivation was carried out at the natural pH of PpB media, 6.1 (for 2% substrate) and 6.5 (for 3% substrate) without any adjustments during the cultivation. An enzyme loading of 5 FPU of commercial cellulase enzyme complex (Cellic Ctec 2) per gram substrate was added (filter sterilized) during the start of the cultivation (time 0). The fermentation was carried out at 35 ± 2 °C for 48 h, with sample collection at every 12 h.

### Analyses

The fungal spore concentration was measured using a Bürker counting chamber. The total sugar, total solid, suspended solid and volatile solids of the samples were measured according to the National Renewable Energy Laboratory (NREL) methods [29, 30]. The total nitrogen content in the samples was determined by Kjeldahl method applying digestion, distillation, and acid–base titration using the InKjel P digestor and the Behrotest S1 distiller (Behr Labor-Technik, Germany). Protein was estimated by multiplying the nitrogen content by the nitrogen-to-protein conversion factor of 6.25 [31]. HPLC (Waters 2695, Waters Corporation, Milford, U.S.A.) was used to analyze the components in all liquid fractions. Acetic acid, ethanol, glucose, glycerol, lactic acid, and xylitol were analyzed using an analytical ion exchange column based on hydrogen ions (Aminex HPX-87H, Bio-Rad, USA) operated at 60 °C with 0.6 mL/min of 5 mM H_2_SO_4_ as eluent. Arabinose, galactose, glucose, mannose, and xylose were analyzed using a lead (II)-based column (HPX-87P, Bio-Rad) with two Micro-Guard Deashing (Bio-Rad) precolumns operated at 85 °C with 0.6 mL/min ultrapure water as eluent. A UV absorbance detector (Waters 2487), operating at 210 nm wavelength, was used in series with a refractive index (RI) detector (Waters 2414). Fructose and sucrose in the liquid samples and starch in the PpB media were measured using assay kits of Megazyme (Ireland). The total chemical oxygen demand (COD) was determined using NANOCOLOR^®^ COD 15000 kit, with the photometric determination of the samples using NANOCOLOR^®^ photometers. The viscosities of the samples were determined using a Brookfield digital viscometer-model DV-E (Chemical Instruments AB, Sweden).

### Statistical analysis

Statistical analysis of the collected data was performed using the software MINITAB^®^ (version 17.1.0). Analyses of variance (ANOVA) of the data used general linear models and a confidence level of 95% was used for all analysis. In the graphs, the average values are presented with an error bar representing one standard deviation. All the results presented in the tables are the average values from duplicate experimental sets and are reported with intervals representing the standard deviation.

## Results and discussion

With an abundance of food and a sedentary lifestyle in many parts of the world there is a need of food that is both nutritious and filling. Consumption of mycoprotein has been shown to improve health in relation to blood cholesterol concentrations, energy intake and glycemic response. Furthermore, it can contribute to satiety and thus decreased energy intake in subsequent meals, something that can increase weight-control [7, 23, 24]. Therefore, mycoprotein can potentially contribute to both prevention and treatment of lifestyle dependent conditions such as obesity and type 2-diabetes; however more research is needed to confirm this. The optimal dose of mycoprotein to boost health also remains unknown. In this study, pea-processing byproduct (PpB) with low nutritional value, obtained as a byproduct of the pea protein isolate process, was used as the substrate for the cultivation of edible ascomycetes and zygomycetes fungi. These fungi are known for their palatability and high protein content, with its potential application as human food components. The results on the characterization of the PpB and the fungal cultivation experiments together with the scale up studies in the airlift reactor are presented and discussed further.

### Pea-processing byproduct (PpB) substrate characterization

The PpB was presented as a white odorless fine powder with the characterization as presented in Table 1. The PpB substrate was characterized to have a total glucan content of 62.38 ± 0.51% (w/w), more than 90% of this being starch. Protein is the second most common component in the material, amounting 18.19 ± 0.33% in dry weight basis. The C:N ratio of the PpB could be determined from the carbohydrate and protein contents as 10.28 ± 0.20. Retrogradation of the starch was observed to determine the best concentration of the substrate PpB in the medium (distilled water) which would not cause the change in the quality of the liquid, while being autoclaved. The PpB concentrations (dry weight) of 1, 2, 3, 4, and 5% (w/v) were tested. For the substrate loadings of 4 and 5%, gelification of the medium was observed. At 3% substrate loading, the gelification was not as clear as in the previous cases although it was still noticed. For 1 and 2% substrate loadings, there was no observed retrogradation of starch.

**Table 1:** Characterization of the pea-processing byproduct (PpB)

Component	Content (% w/w in dry basis)
Protein	18.19 ± 0.33
Ash	2.98 ± 0.03
Moisture	10.54 ± 0.19
Arabinans	2.61 ± 0.06
Xylans	0.00
Galactans	2.30 ± 0.04
Glucans	62.38 ± 0.51
Of which	
Starch	56.34 ± 2.52

Additionally, the viscosity of the PpB suspension was determined. The rheological properties of the cultivation medium have effects on momentum, heat and mass transfer, influencing the fermentation performance. In reactors, the flow properties of the media cause changes in the coalescence of the air bubbles, the bulk mixing, the process control, and the formation of stagnant zones [32]. The 2% PpB suspension was determined to have a viscosity of 1.93 ± 0.15 cP before sterilization. After sterilization in the autoclave, however, the viscosity increased to 10.47 ± 0.12 cP, which means the viscosity increased by 441%. The effect of the viscosity of the medium in the performance of bioreactors generally remains unclear. Some studies associate the increase in the viscosity with the reduction of the liquid turbulence, promoting bubble coalescence and the decrease of the gas holdup. On the other hand, other researchers have found an increased gas holdup in viscous media [33].

### PpB as substrate for efficient fungal growth

**Fig. 1:**
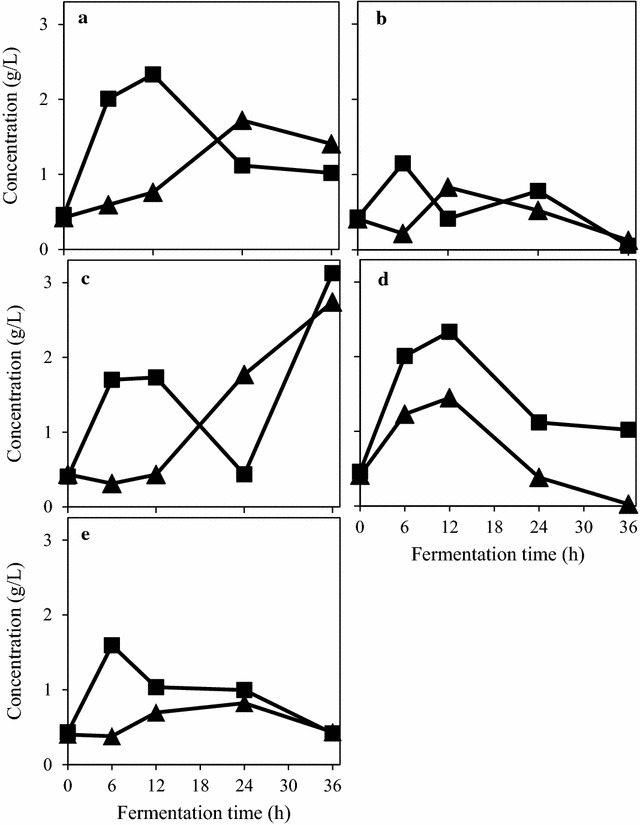
Glucose concentration profile during the filamentous fungal cultivation in 2% (w/v) PpB substrate with no external enzyme supplementation (filled triangles) and with the addition of 150 U/g of α-amylase (filled squares). The figure represents fungal strains *M. purpureus* (**a**); *A. oryzae* (**b**); *F. venenatum* (**c**); *N. intermedia* (**d**); and *R. oryzae* (**e**). Coordinates represent the mean values of duplicate tests; with error bars representing standard deviations omitted due to negligible values

The cultivation of four strains of ascomycetes (*N. intermedia*; *A. oryzae*; *M. purpureus*; and *F. venenatum*) and one of the zygomycetes fungi *(R. oryzae*) was examined in a suspension containing 2% (w/v) PpB substrate using 250 mL Erlenmeyer flasks. The tests were carried out with and without α-amylase addition and in duplicate samples. The effect of the cultivation temperature was not studied in this experimental set. Even without enzyme addition, the ascomycete strains showed a good capacity to hydrolyse starch to glucose (Fig. 1a). *M. purpureus* and *R. oryzae* consumption of glucose exceeded the sugar production rate after 24 h. On the other hand, the sugar consumption rates of *A. oryzae* and *N. intermedia* became higher than the sugar production rate after 12 h. Consumption of glucose by *F. venenatum* was always lower than glucose production. On the other hand, when α-amylase was added (Fig. 1b), *M. purpureus* consumed the glucose at a faster rate than its production after the first 12 h of cultivation. *N. intermedia* presented the same behaviour. *R. oryzae* showed an even higher glucose uptake, overtaking the glucose production rate after the first 6 h of cultivation. Glucose profile during *A. oryzae* and *F. venenatum* cultivation oscillated between increasing and decreasing the glucose concentration in the medium during the cultivation, likely because of different glucose production and glucose uptake rates.

**Fig. 2:**
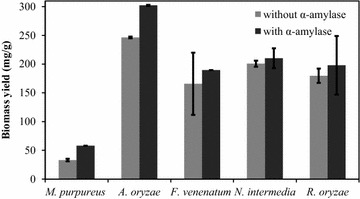
Biomass yield; mg dry fungal biomass per gram of PpB substrate after 36 h of cultivation in 2% w/v PpB medium

*N. intermedia* produced the most ethanol among the microorganisms (4.30 g/L with enzyme addition) while *M. purpureus* cultivation resulted in the lowest final ethanol concentration (0.28 g/L without enzyme addition). *R. oryzae* also converted glucose into lactic acid, yielding 0.66 g/L. α-amylase addition to the media did not result in a significant change in the ethanol and lactic acid production for the ascomycetes strains. However, *R. oryzae* had its ethanol and lactic acid production increased by the addition of the enzyme. Inhibitory effect of these compounds were not considered since the microorganisms have been cultivated in media with concentrations higher than the ones obtained in this study [9, 34]. Biomass production after 36 h is shown in Fig. 2. *M. purpureus* produced significantly less biomass than the other strains (*p* value ≤ 0.031), except *F. venenatum* (*p* = 0.185). *A. oryzae* produced more biomass than *R. oryzae* (*p* = 0.074) and *N. intermedia* (*p* = 0.038). The addition of α-amylase to the medium significantly affected only *A. oryzae* (*p* = 0.003) and *M. purpureus* (*p* = 0.038) growth. When comparing the protein content of the harvested biomass, cultivation of *A. oryzae* resulted in the highest yield of protein per gram of PpB substrate, 0.14 g/g (Table 2).

**Table 2:** Protein yield from the fungal biomass obtained after 36 h cultivation in 2% pea-processing byproduct (PpB) substrate

Enzyme addition	Microorganism	% Protein in dry fungal biomass	Protein yield (g/g PpB substrate)
Without α-amylase	*M. purpureus*	53.61	0.02
	*A. oryzae*	43.13	0.11
	*F. venenatum*	55.28	0.09
	*N. intermedia*	54.53	0.11
	*R. oryzae*	50.03	0.09
With α-amylase	*M. purpureus*	58.66	0.03
	*A. oryzae*	46.36	0.14
	*F. venenatum*	59.75	0.11
	*N. intermedia*	54.11	0.11
	*R. oryzae*	54.79	0.11

**Fig. 3:**
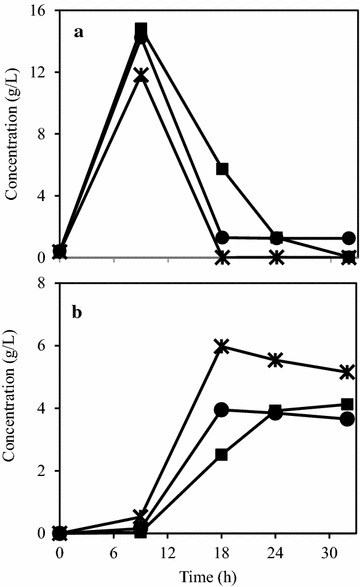
Glucose (**a**) and ethanol (**b**) concentration profiles during the filamentous fungal cultivation in 2% (w/v) PpB substrate. Figure represents fungal strains *A. oryzae* (filled squares); *N. intermedia* (asterisk); and *R. oryzae* (filled circles)

From the previous results, three strains were being considered the most promising ones. *A. oryzae*, *N. intermedia*, and *R. oryzae* were cultivated in the same medium as before (2% PpB substrate), with the addition of the amyloglucosidase enzyme and the cellulase cocktail to test if a higher biomass yield would be obtained. As observed in Fig. 3, *N. intermedia* was most efficient in consuming the glucose, with its concentration reaching zero in about 18 h. Maximum ethanol concentration reaching 5.98 g/L, was also observed with *N. intermedia*. The fungal protein yield obtained for *A. oryzae* and *R. oryzae* was 0.09 g/g of PpB substrate while for *N. intermedia* it was 0.10 g/g.

### Cultivation of *A. oryzae* in the airlift bioreactor

**Fig. 4:**
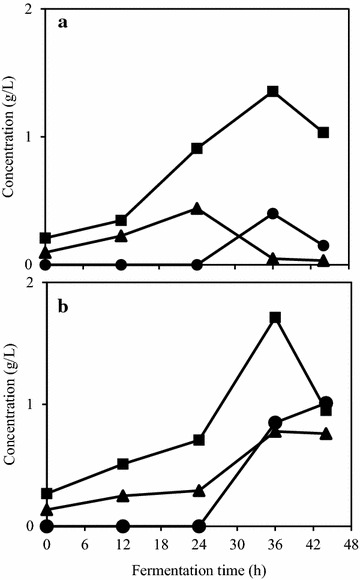
Sugar and ethanol concentration profiles during the cultivation of *A. oryzae* in **a** 2% and **b** 3% (w/v) PpB substrate in an airlift bioreactor. Figure represents glucose (filled squares); other sugars (filled triangles); and ethanol (filled circles). Other sugars are presented in xylose equivalent

*A. oryzae* growth was examined in a bench-scale airlift bioreactor. Since *A. oryzae* is known as an amylase producer [35], for the further experiments the medium was supplemented only with the cellulase cocktail. Four cultivations were run in the bioreactor to test two concentrations of PpB substrate in duplicates: 2 and 3%, resulting in media with pH 6.1 and 6.5 respectively. The results presented in Fig. 4 shows that for both tested scenarios glucose concentration increased up to 36 h. Additionally, 44 h were not enough for the total consumption of the sugar. When the pea processing load was 2%, the other sugars were consumed and at 36 h their concentration was below 0.10 g/L. On the other hand, when the initial load of PpB substrate was 3%, the concentration of the sugars (except glucose) reached the maximum value at 36 h. The ethanol profiles were also divergent, with maximum concentration being reached at 36 h (0.40 g/L) for 2% PpB substrate load and at 44 h (1.01 g/L) for 3% PpB substrate load. The obtained protein yields were 0.26 and 0.13 g/g of PpB substrate for 2 and 3% of the substrate, respectively. The yield for 2% PpB medium is almost twice the value obtained in the shake flask experiments. Moreover, working with a low load of the substrate (and low viscosity) resulted in increased protein production. Airlift bioreactors are known for their capacity to operate using higher aeration rates when compared to traditional stirred fermenters [36]. Moreover, efficient oxygen transfer and mixing are obtained when using airlifts [37]. As a result, cultivation in airlift bioreactor yielded low ethanol concentrations and high fungal protein.

### Process integration at the existing pea-processing industrial facilities

The use of co-products from the industries as important sources of protein for human consumption has been receiving considerable attention. A method of protein production, applicable to a wide range of industrial processes, is to use edible filamentous fungi which could be used as a protein rich meat substitute. Filamentous fungi have been reported as important industrial microorganisms for their capacity to produce numerous metabolites with high market potential [38]. Among these metabolites, the vast assortment of enzymes produced by these microorganisms has special importance. These catalysts are responsible for making filamentous fungi a flexible group able to use a large range of biomaterials as substrate.

**Fig. 5:**
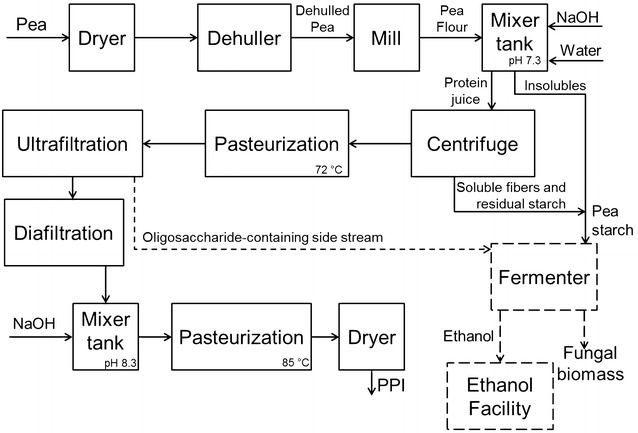
Block flow diagram for the production of pea-protein isolate integrated with the production of vegan-mycoprotein concentrate by filamentous fungi. Adapted from [39]

The scheme of an industrial process for the production of PPI is presented in Fig. 5 [39] with the proposal of integrating the production of fungal biomass for the valorization of the byproduct. The starch and fibers separated from the pea protein would be fed in a bioreactor. For dilution of the solid material, the low-protein content stream from the ultrafiltration step could be used. This stream is sterile and rich in oligosaccharides, which can also be used by the fungi as substrate. Following the same yields of the bench-scale airlift bioreactor, 1 ton of pea-processing byproduct would consume 50 m^3^ of water, and would be predicted to produce 680 kg of *A. oryzae* biomass with 260 kg of pure protein under ideal conditions. The biomass, however, would need to be subjected to a heat treatment to reduce its RNA content, resulting in some loss of protein [14]. Moreover, tests for mycotoxin production should be carried out frequently and, in case of detection, application of corrective methods can affect the productivity [14].

The scenario also includes a system in which the broth containing ethanol could be either sent to a distillation unit or to any adjacent ethanol facilities in the vicinity which contain all the necessary installation for ethanol purification [34]. However, the techno-economic feasibility of using PpB as a potential substrate for edible fungal cultivation for feed or food component needs to be verified in detail and hence open for future studies.

## Conclusion

The pea-processing byproduct was proved to be an efficient medium for the growth of filamentous fungi to produce a vegan-protein concentrate. Fungal biomass with about 46 and 54% protein content was obtained from PpB using edible strains of filamentous fungi, *A. oryzae* and *N. intermedia* respectively. Scaling-up of the process to a 4.5 L bench scale airlift bioreactor improved the *A. oryzae* biomass yield, with a total of 0.26 g of fungal protein per g of PpB. Based on the results obtained, an industrial scenario for the production of vegan-mycoprotein concentrate for human nutrition is proposed as an integrated process to the existing PPI production facilities.
